# Infusion Reactions Associated with the Medical Application of Monoclonal Antibodies: The Role of Complement Activation and Possibility of Inhibition by Factor H

**DOI:** 10.3390/antib7010014

**Published:** 2018-03-14

**Authors:** Tamás Fülöp, Tamás Mészáros, Gergely Tibor Kozma, János Szebeni, Mihály Józsi

**Affiliations:** 1 Nanomedicine Research and Education Center, Semmelweis University, 1089 Budapest, Hungary; tmeszaros@seroscience.com (T.M.); kozmalak@gmail.com (G.T.K.); jszebeni2@gmail.com (J.S.); 2 SeroScience Ltd., 1089 Budapest, Hungary; 3 Department of Nanobiotechnology and Regenerative Medicine, Faculty of Health, Miskolc University, 3515 Miskolc, Hungary; 4 Complement Research Group, Department of Immunology, ELTE Eötvös Loránd University, 1117 Budapest, Hungary; 5 MTA-ELTE Immunology Research Group, Department of Immunology, ELTE Eötvös Loránd University, 1117 Budapest, Hungary

**Keywords:** CARPA, complement, complement activation, factor H, hypersensitivity, infusion reaction, monoclonal antibody therapy, pseudoallergic reaction

## Abstract

Human application of monoclonal antibodies (mAbs), enzymes, as well as contrast media and many other particulate drugs and agents referred to as “nanomedicines”, can initiate pseudoallergic hypersensitivity reactions, also known as infusion reactions. These may in part be mediated by the activation of the complement system, a major humoral defense system of innate immunity. In this review, we provide a brief outline of complement activation-related pseudoallergy (CARPA) in general, and then focus on the reactions caused by mAb therapy. Because the alternative pathway of complement activation may amplify such adverse reactions, we highlight the potential use of complement factor H as an inhibitor of CARPA.

## 1. Introduction: Monoclonal Antibodies and Hypersensitivity Reactions

Monoclonal antibodies (mAbs) are made by identical immune cells that are all clones of a unique parent B cell, and are widely used both in basic research and the therapy of various diseases. For the latter purpose, one of the main goals of scientists became to create “fully” human products to reduce the side effects of humanized or chimeric therapeutic antibodies. These side effects include the induction of hypersensitivity reactions (HSRs), also known as infusion reactions (IRs) [1]. A selected list of anticancer and anti-inflammatory mAbs that cause such HSRs with various incidence and severity is shown in Table 1 [2,3,4,5,6]. 

HSRs have been traditionally categorized in four groups, from I to IV, according to Coombs and Gell. This concept defined Type I reactions as IgE-mediated acute reactions, while the rest of the categories included subacute or chronic immune changes triggered or mediated by IgG, immune complexes, or lymphocytes [7]. However, it has increasingly been recognized that a substantial portion of acute allergic reactions, whose symptoms fit in Coombs and Gell’s Type I category, are actually not initiated or mediated by pre-existing IgE antibodies. These reactions are known to be “pseudoallergic” or “anaphylactoid”. There are estimates that pseudoallergy may represent as high as 77% of all immune-mediated immediate HSRs [8], implying hundreds of thousands of reactions and numerous fatalities every year [9]. Many of these reactions involve the activation of the complement system, an essential humoral arm of innate immunity. Complement activation-related pseudoallergy (CARPA) is linked to adverse events evoked by several liposomal and micellar formulations, nanoparticles, radiocontrast agents, and therapeutic antibodies [9].

Intravenous application of numerous drugs and medical agents, including therapeutic mAbs, enzymes, radiocontrast media, and many other particulate drugs with physical size in the upper nano (10^−8^–10^−7^ m) dimension (nanomedicines), can elicit HSRs with symptoms listed in Table 2. 

## 2. The Consequences of Complement Activation for the Activator and the Host

One of the major tasks of the complement system is to mark and dispose of potentially dangerous particles, such as pathogenic microbes and altered host cells. This is achieved by targeted activation on foreign surfaces as well as on modified host targets, such as apoptotic cells. The classical pathway is activated by immunoglobulins bound to their target antigens, and the classical and lectin complement pathways are activated upon the recognition of certain molecular patterns associated with microbes or altered self, while the alternative pathway is activated constantly at a low rate and in an indiscriminative manner [11]. The activation can result in the deposition of opsonic molecules on the target cells or particles, thus labeling them for phagocytosis and, if not inhibited, allowing the initiation of the terminal pathway that may generate lytic complexes in the target cell’s membrane. The three pathways merge at the activation of the central C3 molecule, which is cleaved into the anaphylatoxin and inflammatory mediator C3a and the larger, opsonic fragment C3b. C3b feeds back to the alternative pathway because it is part of the enzyme complex that cleaves additional C3 molecules. Thus, the alternative pathway can amplify complement activation initiated by any of the three pathways. Importantly, complement regulators expressed in body fluids and on cell surfaces protect the host from bystander damage [11].

Complement activation by liposomes can easily be rationalized on the basis of their resemblance to pathogenic viruses. In fact, both are phospholipid-coated vesicles in the same size range (60–200 nm), with the difference being that liposomes do not express surface proteins as viruses do. In the case of viruses, some of these surface proteins inhibit complement activation just as complement receptor type 1 (CR1), decay accelerating factor (DAF), and membrane cofactor protein (MCP) do on the surface of host blood cells and other cells. One may therefore conclude that liposomal nanomedicines activate complement because the immune system considers them as pathogenic viruses, and liposomes do not have a shield that protects them against complement attack [12]. The mechanism of complement activation by smaller nanoparticles (d < 10 nm), such as PEGylated polyethylene-imine polymers (PEG is polyethylene glycol) [13] or micelles formed from Cremophor EL (CrEL) and other polyethoxylated surfactants (PS-80 and PS-20, also known as Tween-20 and Tween-80) [14] is more difficult to explain. In those cases, complement activation may involve unconventional direct interaction with complement proteins, or, as it was suggested for CrEL, prior interaction with plasma lipoproteins that can lead to the formation of large(r) aggregates [9].

Furthermore, it is already shown in vitro that the aggregation of proteins during the preparation of mAbs can induce the activation of human monocyte-derived dendritic cells as well as T cell responses [15]. Complement activation is also possible in such conditions.

## 3. Therapeutic mAbs, Complement Activation, and CARPA

Antibodies are well known to activate the classical complement pathway upon binding to their target antigen, which allows for the binding of C1q, the recognition molecule of the activation initiator C1 complex, to the Fc part of the antibodies. Therapeutic mAbs may exploit this feature and can be engineered to enhance the effectiveness of the treatment while circumventing certain (e.g., Fc-receptor-mediated) adverse effects [16,17]. 

**Figure 1:**
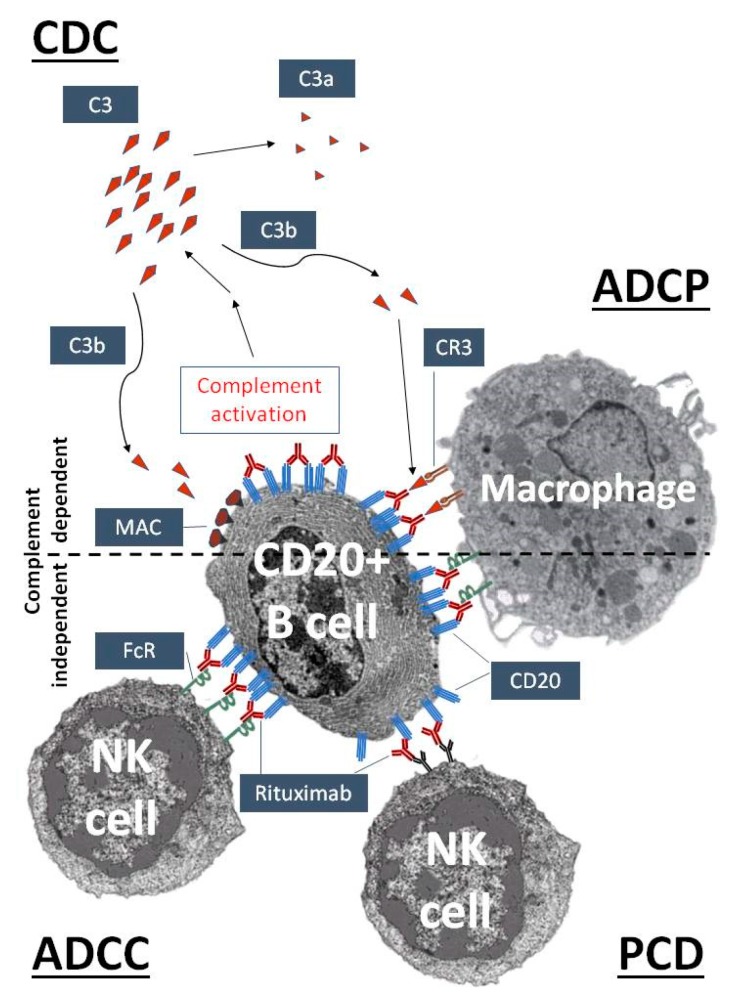
Complement activation as an essential mechanism of the therapeutic action of rituximab, an anti-CD20 antibody. Rituximab recognizes CD20 on the surface of pre- and mature B cells. After binding, the complement activation cascade is initiated by the classical pathway leading to the cleavage of C3 into C3a and C3b. C3b can cause complement-dependent cytotoxicity (CDC) by promoting the assembly of the membrane attack complex (MAC), while complement receptors on phagocytic cells, such as complement receptor type 3 (CR3) on macrophages, can mediate complement-enhanced antibody-dependent cell-mediated phagocytosis (ADCP). Surface-bound rituximab can trigger natural killer (NK) cells and macrophages by complement-independent mechanisms, via antibody-dependent cell-mediated cytotoxicity (ADCC), ADCP and, to a lesser degree, the induction of programmed cell death (PCD).

The role that complement plays in mAb therapy is exemplified well by the prototypic mAb rituximab. Rituximab, a murine-human chimera type anti-CD20, has been used since 1997 in clinical practice to treat malignant and autoimmune disorders related to the disfunction of B cells [18,19]. Besides the direct downregulation of CD20-related cell functions, both complement-dependent and complement-independent immune reactions participate in the elimination of CD20 highly positive B cells (Figure 1). Complement-dependent mechanisms include complement-dependent cytotoxicity (CDC), initiated upon C1q binding, through the classical complement activation cascade [20], and complement-enhanced antibody-dependent cell-mediated phagocytosis (ADCP). The most important complement-independent mechanism is antibody-dependent cell-mediated cytotoxicity (ADCC), which is performed mainly by natural killer (NK) cells (and macrophages). Programmed cell death (PCD) seems to be less important in the case of rituximab, but it may have more prominent role in the action of Type II-anti-CD20 antibodies, like tositumomab and GA101 [18,19]. However, it is likely that the complement-activating capacity of rituximab is also responsible for the high frequency of CARPA associated with this mAb [21].

Human IgG1 and IgG3 are particularly effective at fixing complement to the target cell surface, and many of the currently approved therapeutic mAbs, like rituximab, are indeed of the IgG1 isotype. A variety of cell-based assays have demonstrated the ability of mAbs to recruit complement components in vitro, but the efficiency of CDC to kill tumor cells in vivo is less clear, particularly for solid tumors, in part because tumor cells themselves express membrane-bound complement regulators as well as the soluble regulator factor H [22,23,24]. Since most of these mAbs work against cancer cells with the help of complement activation, a clear distinction has to be made between complement activation on the target cell surface with the help of the cell-bound mAb (i.e., CDC) and adverse hypersensitivity reaction related to complement activation in serum caused by the therapeutic antibody itself. This means that the same mechanisms are involved in the beneficial effects and hypersensitivity.

All currently available or publicly known mAbs can be considered to be potentially direct immunogens, as their molecular size is large enough and their structure is different from endogenous proteins. Despite current efforts to produce highly humanized or “human-like” mAbs, immunogenicity is not yet totally eradicated. Treatment of human patients with mAbs can be associated with the development of specific antibodies against these therapeutic antibodies (anti-drug antibodies, ADAs). These neutralizing ADAs can block the biological activity of the drug either by binding directly to the epitope(s) within their active site, or by steric hindrance due to binding to epitope(s) in close proximity to the active site. The presence of neutralizing ADAs may not result in adverse clinical effect, except that it decreases the efficacy of the therapeutic mAb, requiring its administration at higher doses. Furthermore, the presence of specific ADAs against mAbs can be associated in some cases with hypersensitivity reactions identical to the CARPA phenomenon delineated above for the case of liposomes and other nano-pharmaceuticals. The rare anaphylactic reactions associated with mAbs including cetuximab, infliximab, or basiliximab represent typical CARPA [25].

True allergic reactions, which are mediated by anti-drug IgE, require prior exposure to the mAb and, consequently, do not occur on the first infusion, except in rare cases where patients have pre-existing antibodies that cross-react with the drug. However, pseudoallergic reactions (IgE-independent reactions possibly mediated by direct immune cell and complement activation) and cytokine release syndrome (CRS) both occur primarily on the first infusion of the drug, although they can also occur on subsequent administrations. The symptoms of all three types of immunologically-mediated infusion reactions (IRs) overlap, making it difficult to identify the cause without additional laboratory work [26].

Rituximab and trastuzumab induce the highest incidence of IRs. In general, the incidence of mAb-induced IRs varies from ~15–20% for cetuximab (including 3% more severe, grade 3, and life threatening, grade 4 reactions) and 40% for trastuzumab first infusion (<1% grades 3–4) to 77% for rituximab first infusion (10% grades 3–4). Even after the fourth infusion, 30% of cancer patients react to rituximab, and the incidence of IRs remains 14% after the eighth infusion. Approximately 80% of fatal reactions occur after the first rituximab infusion. The incidence of IRs to the humanized mAb bevacizumab and the fully humanized panitumumab is significantly lower [27].

Thrombocytopenia, neutropenia, and anemia can occur in some patients treated with mAbs as part of anticancer immunotherapy, but the mechanisms of these potentially severe side effects frequently remain unexplored. Interestingly, these symptoms are also characteristic of liposome-induced CARPA. Late-onset neutropenia, especially after rituximab treatment, has been examined in a growing number of reports; however, with each of the three cytopenias seen during mAb therapy, it is frequently unclear whether the depletion of cells is due to an immunological mechanism. Type III hypersensitivities, such as serum sickness-like reactions and vasculitis, are also known to occur in response to mAbs. Some pulmonary events, including mAb-induced lung diseases, are hypersensitivity reactions that result from the interaction of the drug with the immune system and involve drug-specific antibodies or T cells [2]. 

Although it remains to be shown in humans, it is hypothesized that mAbs could stimulate anti-mAb IgGs bound to Fc-gamma-receptors on macrophages, basophils, and neutrophils, triggering the release of platelet-activating factor, as shown in the mouse model of IgG-dependent anaphylaxis [28]. In addition, the complement system could be activated by the formation of large immune complexes, thereby generating anaphylatoxins (C3a and C5a). It is also important to point out that patients with anti-infliximab IgGs are at increased risk of immediate HSRs compared with patients without such antibodies [1]. Thus, in addition to the preferred complement activation induced by the binding of therapeutic mAbs to their targets, complement activation can also arise as a consequence of the binding of naturally forming ADAs against the therapeutic mAbs. The molecular background of mAb-induced CARPA is yet to be studied in more detail.

## 4. Potential Role of Factor H in Mitigating Complement Activation

The use of natural or engineered complement inhibitors may represent an attractive way to prevent CARPA-mediated HSRs. Early approaches used the complement-regulatory domains of the natural complement inhibitor CR1 linked to a myristoyl group that mediated incorporation in liposomal membranes [29]. A recent study suggested that factor H could be also employed to reduce or eliminate complement activation triggered by liposomes, micelles, or therapeutic mAbs [30]. Factor H is the main soluble inhibitor of the alternative pathway and the amplification loop of complement [31,32]. It was shown that liposomal Amphotericin B, CrEL, and rituximab caused less complement activation in serum in vitro when factor H was added to the serum in excess, as compared with the serum without exogenous factor H [30]. Moreover, the artificial inhibitor, recombinant mini-factor H [33], which unites the N-terminal complement-regulatory domains and the C-terminal host surface recognition domains of the natural molecule, was even more effective in inhibiting such complement activation compared with factor H [30]. These data suggest that factor H-based complement inhibition could be a viable strategy to prevent or mitigate CARPA induced by nanomedicines, including therapeutic mAbs.

## 5. Conclusions and Outlook

The prevention of IRs induced by mAbs can be addressed the same way as the prevention of similar adverse reactions occurring upon nanomedicine treatments. The surface modification of liposomes and other therapeutic proteins can lead to prevention of the aggregation of these agents and reduction of immunogenicity and antigenicity. Recently, more and more antibodies and, predominantly, antibody fragments designed for therapeutic purposes use the covalent attachment of polyethylene glycol (PEG). PEGylation generally prolongs the half-life in the circulation and prevents the immunogenicity of many liposomal drugs and mAb molecules [34]. However, in some cases the generation of an IR event could be connected to the presence of PEGylation on the surfaces of liposomes. The formation of anti-PEG IgMs against PEG molecules on liposomes are observed in CARPA studies with animal models [35].

Another possible approach is the administration of complement inhibitors together with the therapeutic agents to reduce the chance of a possible adverse reaction. Even though this could be a good option as a prevention measure, most patients may not even need such an action if they are not prone to IRs, and this approach would just elevate the costs of the therapies. The best scenario would be to pre-screen each patient for proneness to any adverse reaction, using an in vitro test that could predict from a blood sample if any CARPA event could arise during introduction of a therapeutic agent, such as mAbs.

## Figures and Tables

**Table 1:** Information on hypersensitivity reactions to marketed monoclonal antibodies.

Brand Name (Manufacturer)	INN, Isotype (Target Antigen)	Indication	Incidence	Symptoms	References
Anticancer use					
Avastin (Genentech, San Francisco, CA, USA; Roche, Basel, Switzerland)	bevacizumab, humanized IgG1 (VEGF-A)	combination chemotherapy of metastatic colon, lung, and kidney cancer, and glioblastoma	<3%, severe: 0.2%	chest pain, diaphoresis, headache, hypertension, neurologic signs and symptoms, oxygen desaturation, rigors, wheezing	[2,4]
Campath (Genzyme, Cambridge, MA, USA)	alemtuzumab–IH, humanized IgG1κ (CD52 on T and B cells)	B cell chronic lymphocytic leukemia (B-CLL)	4–7%	bronchospasm, chills, dyspnea, emesis, fever, hypotension, nausea, pyrexia, rash, rigors, tachycardia, urticaria	[2,5]
Erbitux (Bristol-Myers Squibb, New York, NY, USA; Eli Lilly, Indianapolis, IN, USA)	cetuximab, chimeric IgG1κ (EGFR)	metastatic colorectal cancer, head and neck cancer, squamous cell carcinomas	<3%, fatal < 0.1%	anaphylaxis, angioedema, bronchospasm, cardiac arrest, chills, dizziness, dyspnea, fever, hoarseness, hypotension, pruritus, rash, rigor, stridor, urticaria, wheezing	[1,2,3]
Herceptin (Genentech, San Francisco, CA, USA)	trastuzumab, humanized IgG1κ (EGFR receptor 2, HER2/neu/erbB2)	metastatic breast and gastric cancer	<1%	asthenia, bronchospasm, chills, death within hours, dizziness, dyspnea, further pulmonary complications, headache, hypotension, hypoxia, nausea, pain, rash, severe hypotension, vomiting	[1,2,3]
Rituxan (Genentech, San Francisco, CA, USA)	rituximab, chimeric IgG1κ (CD20 on B cells)	B cell leukemias, rheumatoid arthritis and non-Hodgkin’s B-cell lymphoma	>80%, severe: <10%	ARDS, bronchospasm, cardiogenic shock, flushing, hypotension, hypoxia, itching, myocardial infarction, pain (at the site of the tumor), pulmonary infiltrates, runny nose, swelling of the tongue or throat, ventricular fibrillation, vomiting	[1,2,3,6]
Anti-inflammatory use					
Remicade (Janssen Biotech. Inc., Horsham, PA, USA)	infliximab, chimeric IgG1κ (TNF alpha)	Crohn’s disease, rheumatoid arthritis, spondylitis ankylopoetica, arthritis psoriatica, ulcerative colitis	18%	bronchospasm, laryngeal edema, pharyngeal edema, dyspnea, hypotension, urticaria, serum sickness-like reactions	[3]
Xolair (Genentech, San Francisco, CA, USA)	omalizumab, humanized IgG4 (IgE)	atopia, asthma	39%, Severe: 0.2%	anaphylaxis, bronchospasm, hypotension, syncope, urticaria, and/or angioedema of the throat or tongue, delayed anaphylaxis (with onset two to 24 h or even longer) beyond one year after beginning regularly administered treatment	[1]

INN: international nonproprietary names; ARDS: acute respiratory distress syndrome.

**Table 2:** Symptoms of pseudoallergy. The most life-threatening symptoms are highlighted in bold [10]. Reprinted from Molecular Immunology, Vol. 61, Szebeni J., Complement activation-related pseudoallergy: A stress reaction in blood triggered by nanomedicines and biologicals, Pages 163–173, Copyright (2014), with permission from Elsevier.

Cardiovascular	Broncho-Pulmonary	Hematological	Mucocutaneous	Gastrointestinal	Neuro-Psycho-Somatic	Systemic
Angioedema	Apnea	Granulopenia	Cyanosis	Bloating	Back pain	Chills
Arrhythmia	Bronchospasm	Leukopenia	Erythmea	Cramping	Chest pain	Diaphoresis
**Cardiogenic shock**	Coughing	Lymphopenia	Flushing	Diarrhea	Chest tightness	Feeling of warmth
Edema	Dyspnea	Rebound leukocytosis	Nasal congestion	Metallic taste	Confusion	Fever
Hypertension	Hoarsness	Rebound granulocytosis	Rash	Nausea	Dizziness	Loss of consciousness
Hypotension	Hyperventillation	Trombocytopenia	Rhinitis	Vomiting	Feeling of imminent death	Rigors
Hypoxia	Laryngospasm		Swelling		Fright	Sweating
**Myocardial infarction**	Respiratory distress		Tearing		Headache	Wheezing
Tachycardia	Shortness of breath		Urticaria		Panic	
**Ventricular fibrillation**	Sneezing					
Syncope	Stridor					
